# Mitochondrial Dysfunction and Changes in High-Energy Compounds in Different Cellular Models Associated to Hypoxia: Implication to Schizophrenia

**DOI:** 10.1038/s41598-019-53605-4

**Published:** 2019-12-02

**Authors:** Luiz Felipe Souza e Silva, Mariana Dutra Brito, Jéssica Mayumi Camargo Yuzawa, Tatiana Rosado Rosenstock

**Affiliations:** Santa Casa de São Paulo School of Medical Sciences, São Paulo, Brazil

**Keywords:** Cellular neuroscience, Schizophrenia, Energy metabolism

## Abstract

Schizophrenia (SZ) is a multifactorial mental disorder, which has been associated with a number of environmental factors, such as hypoxia. Considering that numerous neural mechanisms depends on energetic supply (ATP synthesis), the maintenance of mitochondrial metabolism is essential to keep cellular balance and survival. Therefore, in the present work, we evaluated functional parameters related to mitochondrial function, namely calcium levels, mitochondrial membrane potential, redox homeostasis, high-energy compounds levels and oxygen consumption, in astrocytes from control (Wistar) and Spontaneously Hypertensive Rats (SHR) animals exposed both to chemical and gaseous hypoxia. We show that astrocytes after hypoxia presented depolarized mitochondria, disturbances in Ca^2+^ handling, destabilization in redox system and alterations in ATP, ADP, Pyruvate and Lactate levels, in addition to modification in NAD^+^/NADH ratio, and *Nfe2l2* and *Nrf1* expression. Interestingly, intrauterine hypoxia also induced augmentation in mitochondrial biogenesis and content. Altogether, our data suggest that hypoxia can induce mitochondrial deregulation and a decrease in energy metabolism in the most prevalent cell type in the brain, astrocytes. Since SHR are also considered an animal model of SZ, our results can likewise be related to their phenotypic alterations and, therefore, our work also allow an increase in the knowledge of this burdensome disorder.

## Introduction

Schizophrenia (SZ) is a multifactorial mental disorder that is related to several hypotheses, including the neurodevelopment theory^1,2^. According to this concept, modifications in the brain machinery, caused by combined environmental factors at critical times, such as pregnancy^2,3^, could lead to changes in brain anatomy and dynamics and, consequently, in its function^1^. One of the main environmental factors related to SZ in clinical practice is hypoxia^4–6^. In 2002, Tyrone and colleagues reported abnormalities in brains from SZ patients that were exposed to intrauterine hypoxia from obstetric complications^6^. Moreover, it is known that intrauterine hypoxia seems to contribute to a reduction in the gray matter and an enlargement in the lateral ventricular zone^3,6^. Interestingly, studies have also shown that hypertension during pregnancy was well documented in records of women that become mothers of schizophrenic patients^7–9^. In agreement, a study designed by Mayoral and collaborators using rodents showed that exposure to chronic hypoxia may lead to loss of tissue volume, decreased myelination and increased ventricles^10^. Surprisingly, many of these abnormalities are also observed schizophrenic patients brains^11–13^.

Although hypoxia contributes to the occurrence of SZ^4,9,14^, it is necessary to investigate the role of mitochondria under this condition, in order to understand how brain cells could adapt and provide energy even with low oxygen voltages. Since mitochondria rely on O_2_ consumption for ATP production^15^, one might speculate that hypoxic conditions could, in turn, negatively affect all ATP-dependent processes contributing to SZ development^16^. Indeed, mitochondria dysfunction seems to play a crucial role in the SZ’s neurobiology, since it was already demonstrated changes in cytochrome C release, mitochondrial translocases expression and lipid peroxidation^17–20^ in addition to changes in genes associated to mitochondrial maintenance, volume, density, dynamics and metabolism^19,21–32^. Therefore, in the present work, we evaluated in astrocytes from Spontaneously Hypertensive Rats (SHR animals) (model of intrauterine hypoxia) and astrocytes exposed to chemical and gaseous hypoxia functional parameters related to mitochondrial function, namely calcium handling, mitochondrial membrane potential, redox homeostasis, oxygen consumption and ATP, ADP, Pyruvate, Lactate and NAD^+^/NADH levels, in addition to gene expression and protein levels related to mitochondrial metabolism. Astrocytes are a very important cell type because they not only give support for neurons, but also play an important role in neurodevelopment^33–35^ since they are involved in neuronal formation and development, orientation and migration of developing axons^34,36^.

Overall, our data reveal that astrocytes submitted to hypoxia have alterations in Ca^2+^ handling, mitochondrial Ca^2+^ uptake and mitochondrial membrane potential, besides an augmentation of redox homeostasis. Concomitantly, intrauterine and chemical hypoxia induce changes in ATP, ADP, Pyruvate and Lactate levels and NAD^+^/NADH ratio, despite an intensification of mitochondrial biogenesis and mitochondrial content.

## Results

### Reduction of astrocytes viability after chemical and gaseous hypoxias

**Figure 1:**
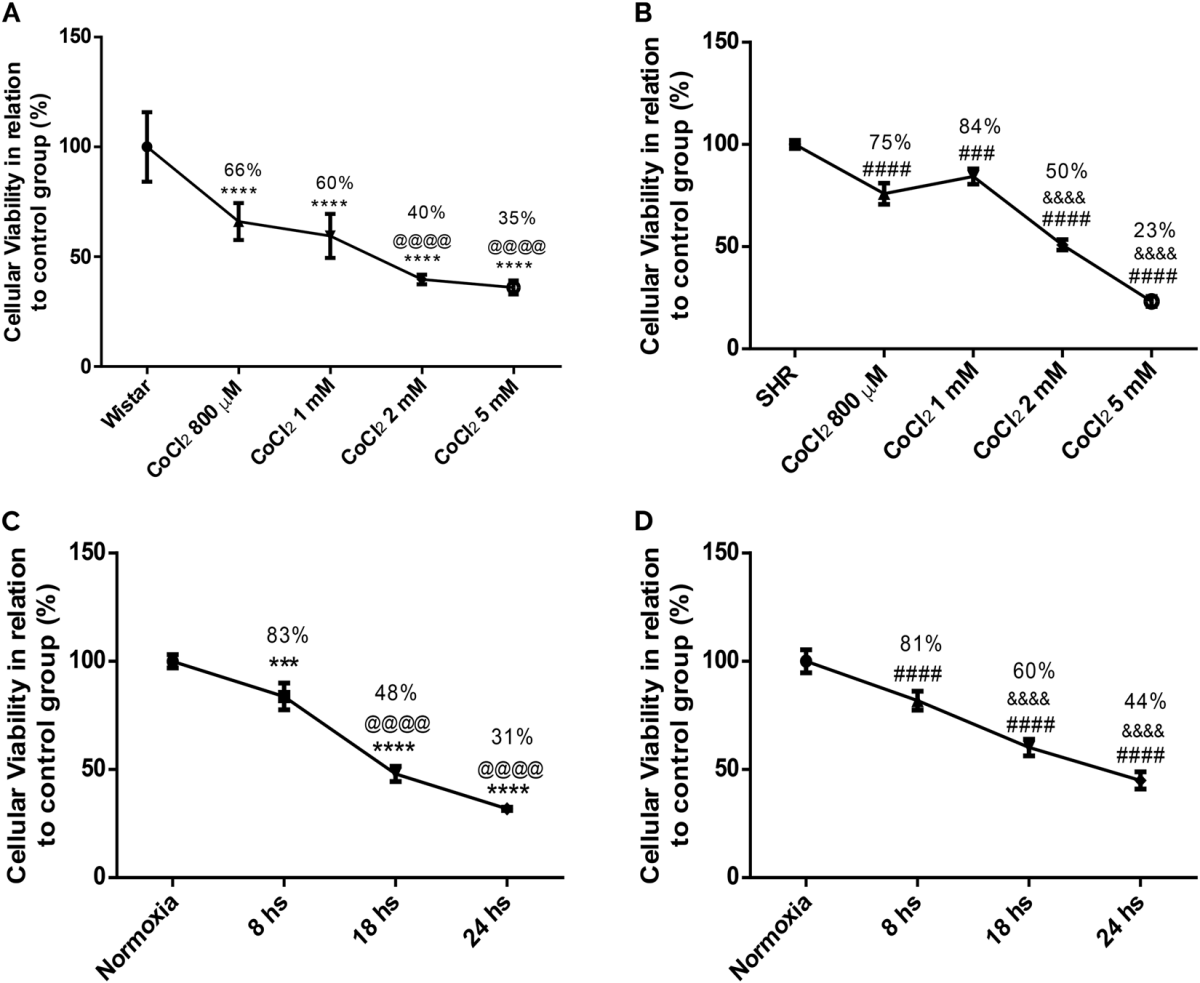
Cellular viability reduction in Wistar (**A**,**C**) and SHR (**B**,**D**) astrocytes after exposure to chemical and gaseous hypoxia. Cells were exposed to CoCl_2_ (800 µM, 1 mM, 2 mM and 5 mM for 24 hrs) and gaseous hypoxia (8, 18 and 24 hrs) and it was performed MTT test. Data is represented by mean ± SD, and it was normalized as percentage of control group (N = 4, in duplicates). Statistical analysis was performed using One-Way ANOVA followed by *post-hoc* Duncan. It was considered significant p < 0.05; ***p < 0.0001 and ****p < 0.00001, in relation to Wistar (both without treatment and normoxia group); ^@@@@^p < 0.00001, in relation to Wistar cells exposed to both CoCl_2_ 800 µM and 8 hrs of hypoxia; ^###^p < 0.0001 and ^####^p < 0.00001, in relation to SHR (both without treatment and normoxia group); ^&&&&^p < 0.00001, in relation to SHR cells exposed to CoCl_2_ 800 µM and 1 mM, and 8 hrs of hypoxia.

To verify the effects of hypoxia on cellular viability submitted to various stimuli, we performed MTT assay. As shown in Fig. 1A, there is a dose-dependent reduction on the viability of Wistar astrocytes after CoCl_2_ treatments (800 µM, 1 mM, 2 mM and 5 mM, 24 hours) in relation to untreated/control group. The same effect was demonstrated in astrocytes from SHR animals (Fig. 1B). In Fig. 1C,D, we can observe that there is a time-dependent reduction of astrocytes survival from either Wistar or SHR animals after exposure to gaseous hypoxia (for 8, 18 and 24 hours).

### Induction of Hif1α pathway in astrocytes submitted to chemical and intrauterine hypoxias

**Figure 2:**
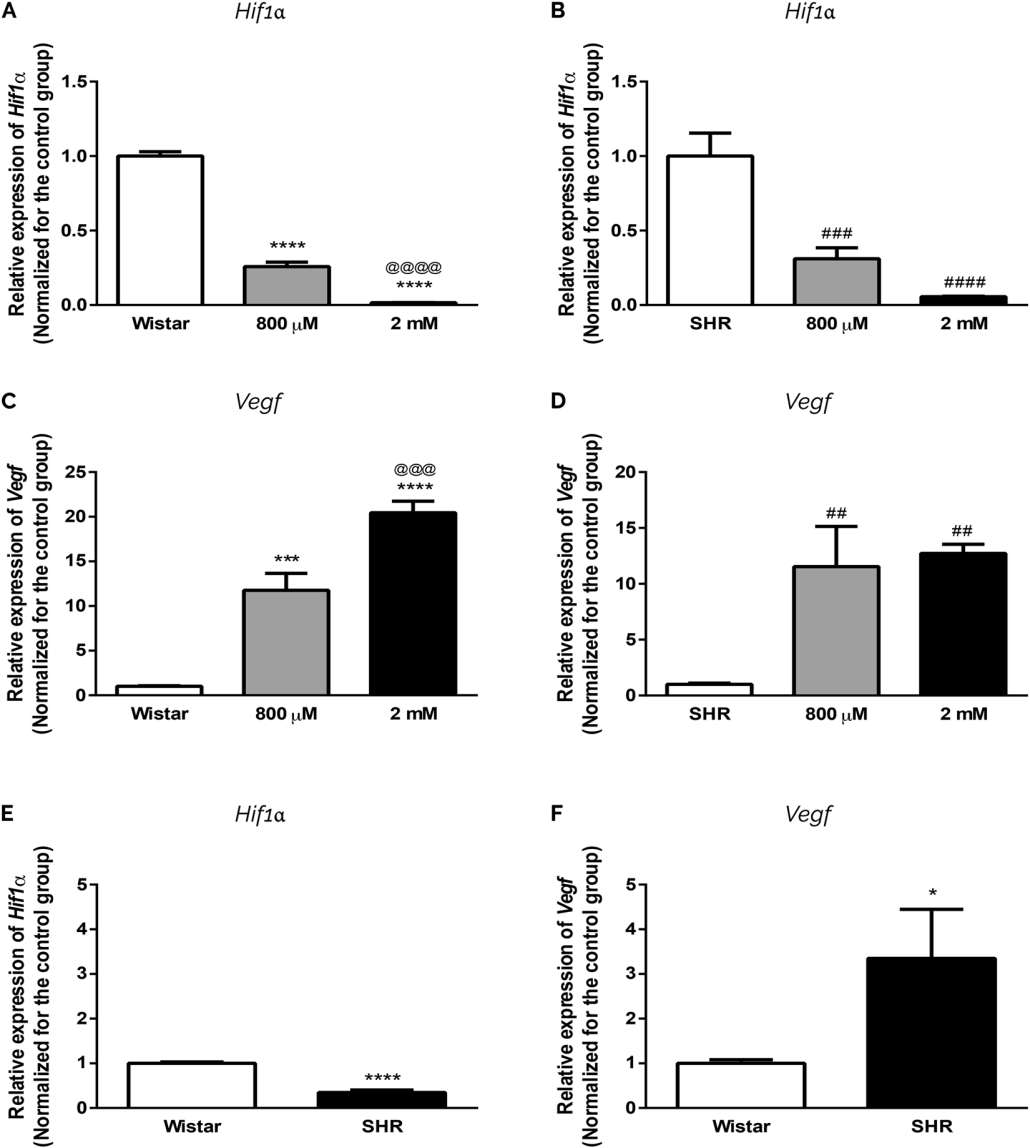
Induction of *Hif1α* pathway in astrocytes submitted to intrauterine and chemical hypoxia. Astrocytes from both groups (Wistar: **A**,**C**; SHR: **B**,**D**) were exposed to 800 µM and 2 mM of CoCl_2_ for 24 hrs. Expression of *Hif1α* (**A**,**C**,**E**) and *Vegf* (**B**,**D**,**F**) is in relation to *βActin*. Data in graphs is the mean ± SD and the results were normalized as percentage of control group (N = 3, in duplicates). Statistical analysis was performed using One-Way ANOVA with *post-hoc* Duncan and Student’s t Test. It was considered significant, p < 0.05; *p < 0.01, ***p < 0.0001 and **** < 0.00001, in relation to Wistar group; ^@@@^p < 0.0001 and ^@@@@^p < 0.00001, in relation to Wistar 800 µM group; ^##^p < 0.001, ^###^p < 0.0001 and ^####^p < 0.0001, in relation to SHR group.

To get evidences regarding the induction of hypoxia pathway, we investigated *Hif1α* and *Vegf* expression^35,37–39^. We demonstrated that CoCl_2_ leads to a significant diminishment in *Hif1α* expression in both Wistar and SHR astrocytes (Fig. 2A,B). However, CoCl_2_ treatment induces a significant augmentation in the expression of *Vegf* in Wistar astrocytes in a dose-dependent manner; the same happens in SHR group (Fig. 2C,D).

Comparing the expression of *Hif1α* between untreated Wistar and SHR astrocytes, we also observe a significant decrease in *Hif1α* expression in cells exposed to intrauterine hypoxia (Fig. 2E). Concomitantly, and as expected, there is a significant increase in *Vegf* expression in SHR astrocytes (Fig. 2F).

### Intrauterine, chemical and gaseous hypoxias induce changes in Ca^2+^ handling and mitochondrial Ca^2+^ uptake

**Figure 3:**
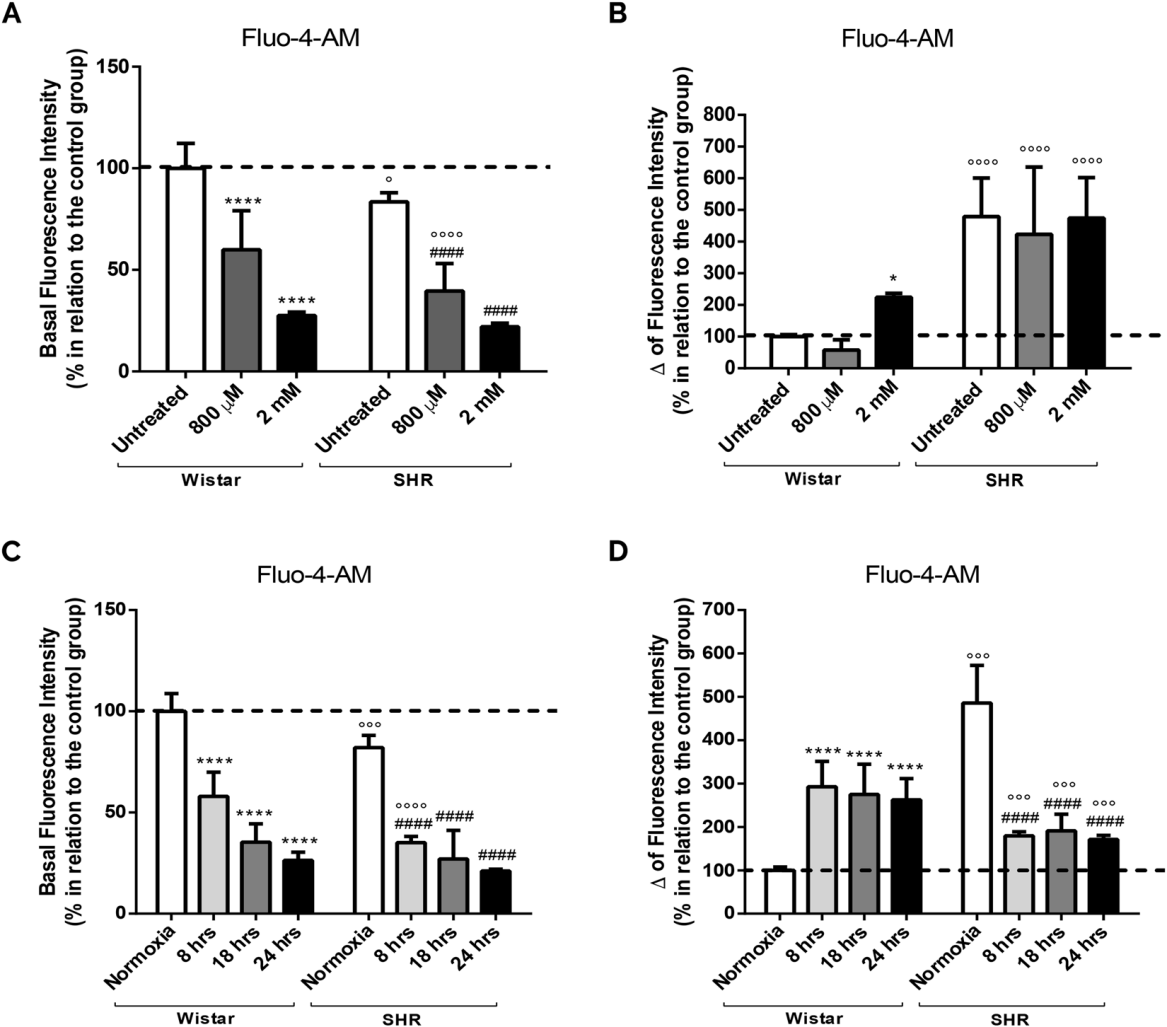
Reduction of cytosolic Ca^2+^ after chemical, gaseous and intrauterine hypoxia and increased calcium uptake by SHR mitochondria. Astrocytes from both groups (Wistar and SHR) were exposed to CoCl_2_ (800 µM and 2 mM, for 24 hrs) and gaseous hypoxia (for 8, 18 and 24 hrs). The histograms represent the basal level of cytosolic Ca^2+^ after incubation with Fluo-4-AM (10 µM, 1 hr) (**A**,**C**) and after stimulation with FCCP (5 µM) (Δ of fluorescence intensity) (**B**,**D**). Data is represented by mean ± SD, and the results were normalized as percentage of control group (N = 4, in duplicates). Statistical analysis was performed using Two-Way ANOVA followed by *post-hoc* Duncan. It was considered significant p < 0.05; *p < 0.01 and ****p < 0.00001, in relation to untreated and normoxia Wistar group; ^####^p < 0.00001, in relation to untreated and normoxia SHR group; ^°^p < 0.01, °°°p < 0.0001 and °°°°p < 0.00001, in relation to the respective Wistar group.

Because mitochondrial function and ATP synthesis relies on oxidative phosphorylation capacity^40^, and modification in the transport of electron and the proton motive force can affect the transport of ions, such as Ca^2+^ ^41^, we evaluated the Ca^2+^ homeostasis (Fig. 3). We can notice that astrocytes from SHR present lower cytosolic Ca^2+^ level in comparison to Wistar’s (untreated groups) (Fig. 3A). Curiously, there is a significant reduction in cytosolic Ca^2+^ level, in relation to untreated cells, after CoCl_2_ exposure in both groups as well (Fig. 3A). In the presence of FCCP, the results show a 5-fold increase in cytosolic Ca^2+^ level in untreated SHR astrocytes (Fig. 3B), suggesting that mitochondria from SHR can uptake more Ca^2+^. Chemical hypoxia also induced a significant release of Ca^2+^ to cytosol in Wistar astrocytes, but only when CoCl_2_ was added at 2 mM (intense hypoxia) (Fig. 3B). On the contrary, SHR astrocytes exposed to chemical hypoxia (800 µM and 2 mM) does not increase Ca^2+^ uptake by mitochondria (Fig. 3B).

In order to compare the effect of CoCl_2_ treatment with the absence of oxygen, we decided to induce gaseous hypoxia. Corroborating our previous data, there is a reduction in the cytosolic Ca^2+^ in untreated SHR astrocytes when compared to Wistar’s (Fig. 3C). Interestingly, gaseous hypoxia also induces a decrease in cytosolic Ca^2+^ in both Wistar and SHR groups (Fig. 3C). Still, when astrocytes are challenged with FCCP, we get not only an increase in the fluorescent signal in untreated SHR astrocytes when compared to Wistar astrocytes, but also an augmentation in both groups after gaseous hypoxia (Fig. 3D). Surprisingly, SHR astrocytes release less Ca^2+^ to cytosol than Wistar astrocytes. These records suggest that gaseous hypoxia does not induce Ca^2+^ uptake by SHR mitochondria.

### Mitochondria depolarization after intrauterine, chemical and gaseous hypoxias

**Figure 4:**
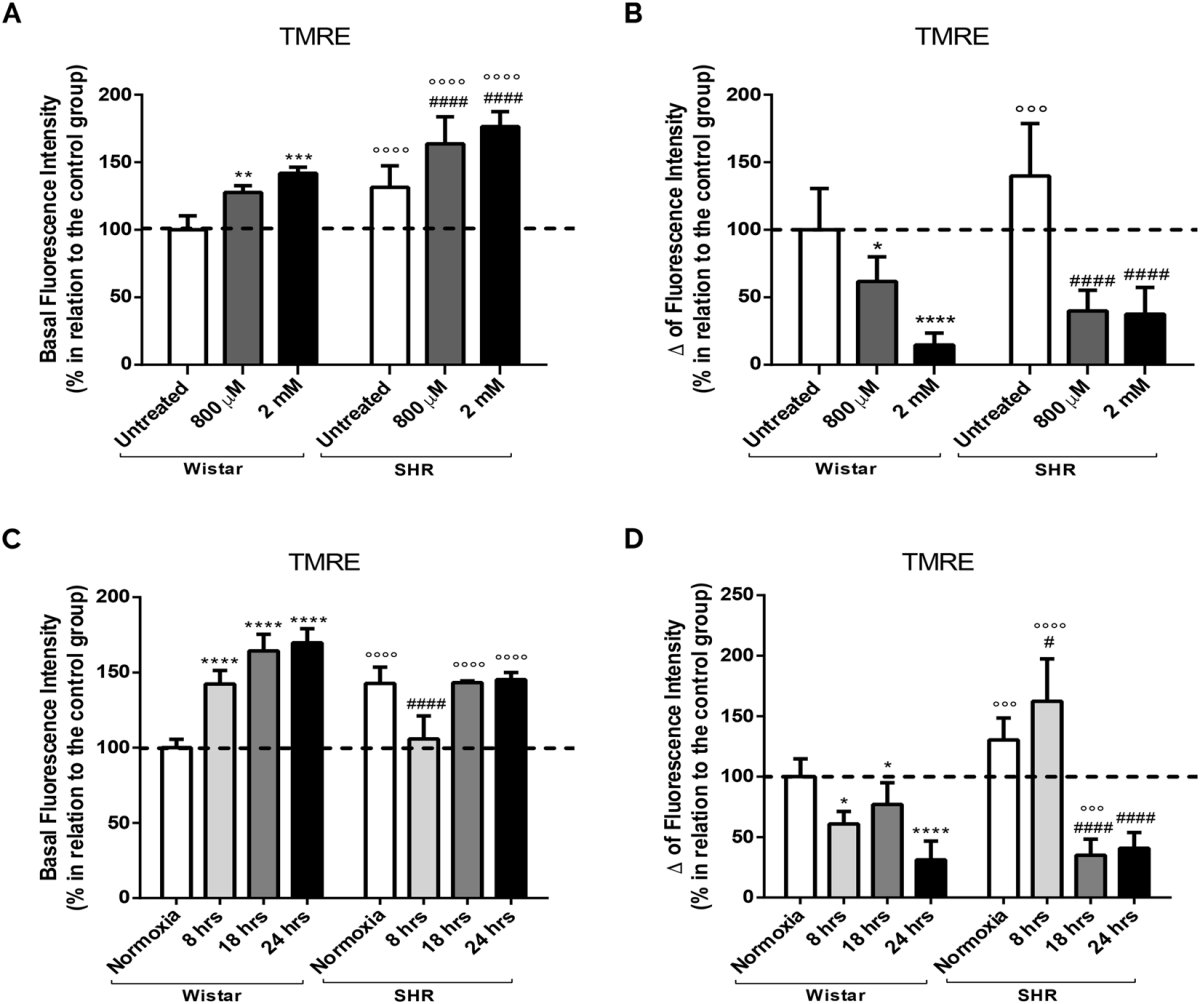
Mitochondrial membrane potential depolarization after chemical, gaseous and intrauterine hypoxia. Astrocytes from both groups (Wistar and SHR) were exposed to CoCl_2_ (800 µM and 2 mM, for 24 hrs) and gaseous hypoxia (for 8, 18 and 24 hrs). The histograms represent the basal level of TMRE fluorescence (500 nM, 1 hr) (**A**,**C**) and after stimulation with FCCP (5 µM) (Δ fluorescence intensity) (**B**,**D**). Data is represented by mean ± SD, and the results were normalized as percentage of control group (N = 4, in duplicates). Statistical analysis was performed using Two-Way ANOVA followed by *post-hoc* Duncan. It was considered significant p < 0.05; *p < 0.01, ***p < 0.0001 and ****p < 0.00001, in relation to untreated and normoxia Wistar group; ^####^p < 0.00001, in relation to untreated and normoxia SHR group; °°°p < 0.0001 and °°°°p < 0.00001, in relation to the respective Wistar group.

To verify if hypoxia and mitochondrial Ca^2+^ uptake was influencing mitochondrial membrane potential, cells were loaded with TMRE. SHR astrocytes show a significant increase in cytosolic TMRE in comparison to untreated Wistar astrocytes (Fig. 4A). Similarly, CoCl_2_ also induce a significant increase in cytosolic TMRE (Fig. 4A), meaning that less TMRE are being uptake by mitochondria after intrauterine and chemical hypoxia.

Thus, in order to check this hypothesis, all cells were challenged with FCCP. Figure 4B shows that there is an increase in TMRE cytosolic fluorescence in SHR astrocytes in relation to untreated Wistar, indicating a greater capacity of SHR mitochondria to TMRE retain. Curiously, after CoCl_2_ treatment, astrocytes from both Wistar and SHR animals show a lower fluorescent signal (Fig. 4B), suggesting a massive mitochondrial depolarization.

After SHR and Wistar astrocytes were exposed to 0% of O_2_, we also observe an increase in cytosolic TMRE signal in Wistar astrocytes in relation to normoxia, but there is a reduction on the TMRE fluorescence in SHR astrocytes after 8 hours and no differences is found after 18 hours and 24 hours (Fig. 4C). After FCCP challenge, we can observer that there is less TMRE being sequestered by Wistar astrocytes mitochondria; the same result is seen in SHR astrocytes after 18 and 24 hours (Fig. 4D). Curiously, after 8 hours of lower O_2_, FCCP induces a higher release of mitochondrial TMRE to cytosol, suggesting that mild gaseous hypoxia, on the other hand, can lead to a greater uptake of calcium by mitochondria.

### Increased ROS production in astrocytes exposed to intrauterine, chemical and gaseous hypoxias

**Figure 5:**
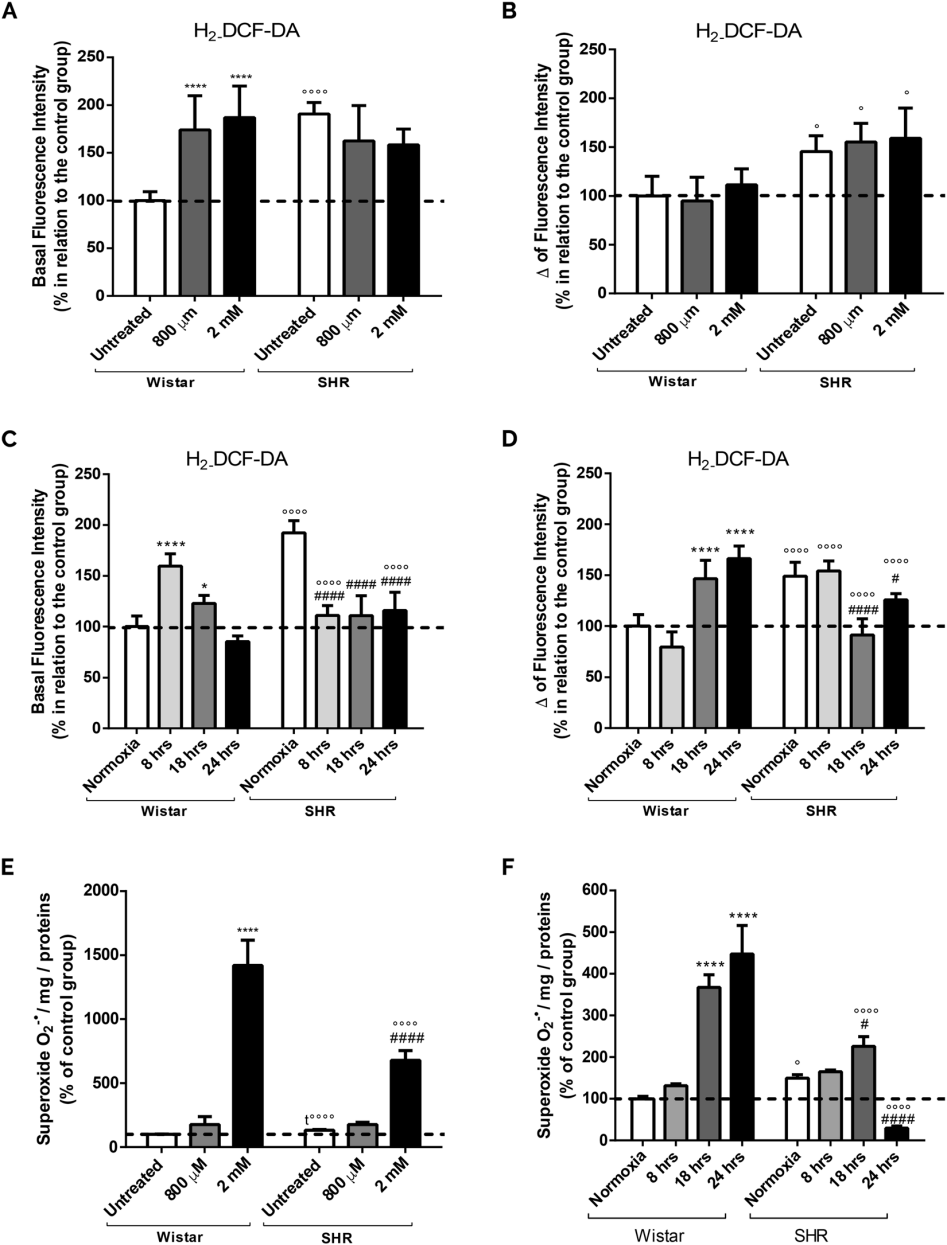
Augmentation of oxidative stress after chemical, gaseous and intrauterine hypoxia. Astrocytes from both groups (Wistar and SHR) were exposed to CoCl_2_ (800 µM and 2 mM, for 24 hrs) and gaseous hypoxia (8, 18 and 24 hrs). Representative histograms of basal H_2_DCF-DA (20 µM for 30 min) fluorescence (**A**,**C**), and after stimulation with FCCP (5 µM) (Δ fluorescence intensity) (**B**,**D**). (**E**,**F**) Representative histograms of the kinetic slope of the O_2_^°−^ levels after chemical (**E**) and gaseous (**F**) hypoxia in cells loaded with Mitosox (5 µM, 10 min). Data is represented by mean ± SD, and the results were normalized as percentage of control group (N = 4, in duplicates). Statistical analysis was performed using Two-Way ANOVA followed by *post-hoc* Duncan or Student’s t Test. It was considered significant p < 0.05; *p < 0.01 and ****p < 0.00001 in relation to untreated and normoxia Wistar group; ^#^p < 0.01 and ^####^p < 0.00001, in relation to untreated and normoxia SHR group; °p < 0.01, °°°°p < 0.00001 and t°°°°p < 0.00001, in relation to the respective Wistar group.

We further decided to investigate whether redox homeostasis was also disturbed in our cellular systems. There is a significant increase in ROS (indicated by CM-H_2_DCFDA fluorescence) in untreated SHR astrocytes in relation to Wistar cells (Fig. 5A), indicating that intrauterine hypoxia is able to induce a significant increment in ROS production. However, CoCl_2_ treatment just leads to a significant ROS augmentation in Wistar astrocytes (Fig. 5A). Our data may be related to the fact that astrocytes from SHR animals already present an increase in ROS.

To check an additional increase in ROS, we performed the experiments in the presence of FCCP (Fig. 5B). We get no difference in Wistar or SHR group between treatments, but there is a significant increment in SHR astrocytes fluorescence signal only when compared to the respectively Wistar treated group. This result indicates that intrauterine and chemical hypoxias promote an increase in ROS production that is not affected by further hypoxic stimuli.

Concerning to gaseous hypoxia, as shown in Fig. 5C, there is a significant ROS production in astrocytes from SHR strain when compared to Wistar group (normoxia) (Fig. 5A); data resemble those with CoCl_2_ treatment. We also detect that Wistar astrocytes exposed to gaseous hypoxia (8 and 18 hours) have an increase in the basal fluorescence in comparison to normoxia group. On the other hand, astrocytes from SHR animals present a significant reduction in ROS production when exposed to gaseous hypoxia in comparison to normoxia condition. In the presence of FCCP, we observe an increase in DCF signal in Wistar astrocytes after 18 and 24 hours of hypoxia (Fig. 5D). Moreover, in the SHR group, the opposite effect is detected, meaning that FCCP does not induce any increase in ROS after hypoxia.

In order to identify superoxide (O_2_^°−^) formation, cells were loaded with Mitosox. Figure 5E shows a significant intensification in the fluorescent slope in SHR astrocytes when compared to Wistar cells (untreated). Additionally, there is a significant increase in O_2_^°−^ levels in Wistar and SHR astrocytes after intense chemical hypoxia compared to its respectively untreated group (Fig. 5E). In agreement, an intense gaseous hypoxia (18 hours) also induces a significant enhancement of O_2_^°−^ in astrocytes from Wistar and SHR animals (Fig. 5F). Strangely, after 24 hours of 0% of O_2_, we have a decrease in the fluorescence signal in SHR astrocytes when compared to SHR.

### Decreased antioxidant defense and reduced expression of key metabolic gene regulators upon intense chemical hypoxia

**Figure 6:**
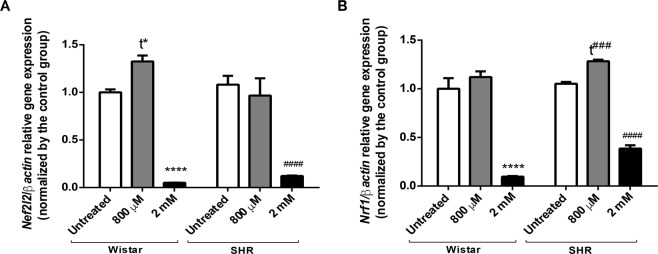
Changes in *Nfe2l2* and *Nrf-1* expression after chemical hypoxia. Relative expression of *Nfe2l2* (**A**) and *Nrf-1* (**B**) in astrocytes from both Wistar and SHR animals treated with CoCl_2_ (800 µM and 2 mM) for 24 hrs. Data is represented by mean ± SD. Results were normalized as percentage of control group (N = 3, in duplicates). Statistical analysis was performed using Two-Way ANOVA followed by *post-hoc* Duncan or Student’s t Test. It was considered significant p < 0.05; ****p < 0.00001 and t* = p < 0.01, in relation to untreated Wistar group; ^####^p < 0.00001, t^###^ = p < 0.0001, in relation to untreated SHR group.

Because redox homeostasis is deregulated after hypoxia, we further investigated *Nfe2l2* expression (Fig. 6A). Curiously, there is no significant change in its expression in SHR astrocytes when compared to astrocytes from Wistar rats. However, there is a significant decline in *Nfe2l2* expression after intense chemical hypoxia in both groups. Interestingly, we verify an augmentation of *Nfe2l2* expression when Wistar astrocytes are exposed to 800 µM of CoCl_2_, suggesting an increase in antioxidant defense after moderate hypoxia. This outcome strongly suggests that a severe chemical hypoxia could induce higher levels of ROS, which in turn leads to a significant decrease in *Nef2l2* expression^42–44^.

Concerning to *Nrf-1*^45^ expression, there is no differences between Wistar and SHR untreated groups. As occurred with *Nfe2l2* expression, *Nrf-1* expression diminishes after intense hypoxia (Fig. 6B). On the contrary, there is a significant increase in *Nrf-1* expression after mild hypoxia in SHR cells, which may be related to a possible adaptation to moderate hypoxia, where ROS levels are increased and could be able to stimulate *Nrf1* expression.

### Enhancement of mitochondrial biogenesis and content in SHR astrocytes

**Figure 7:**
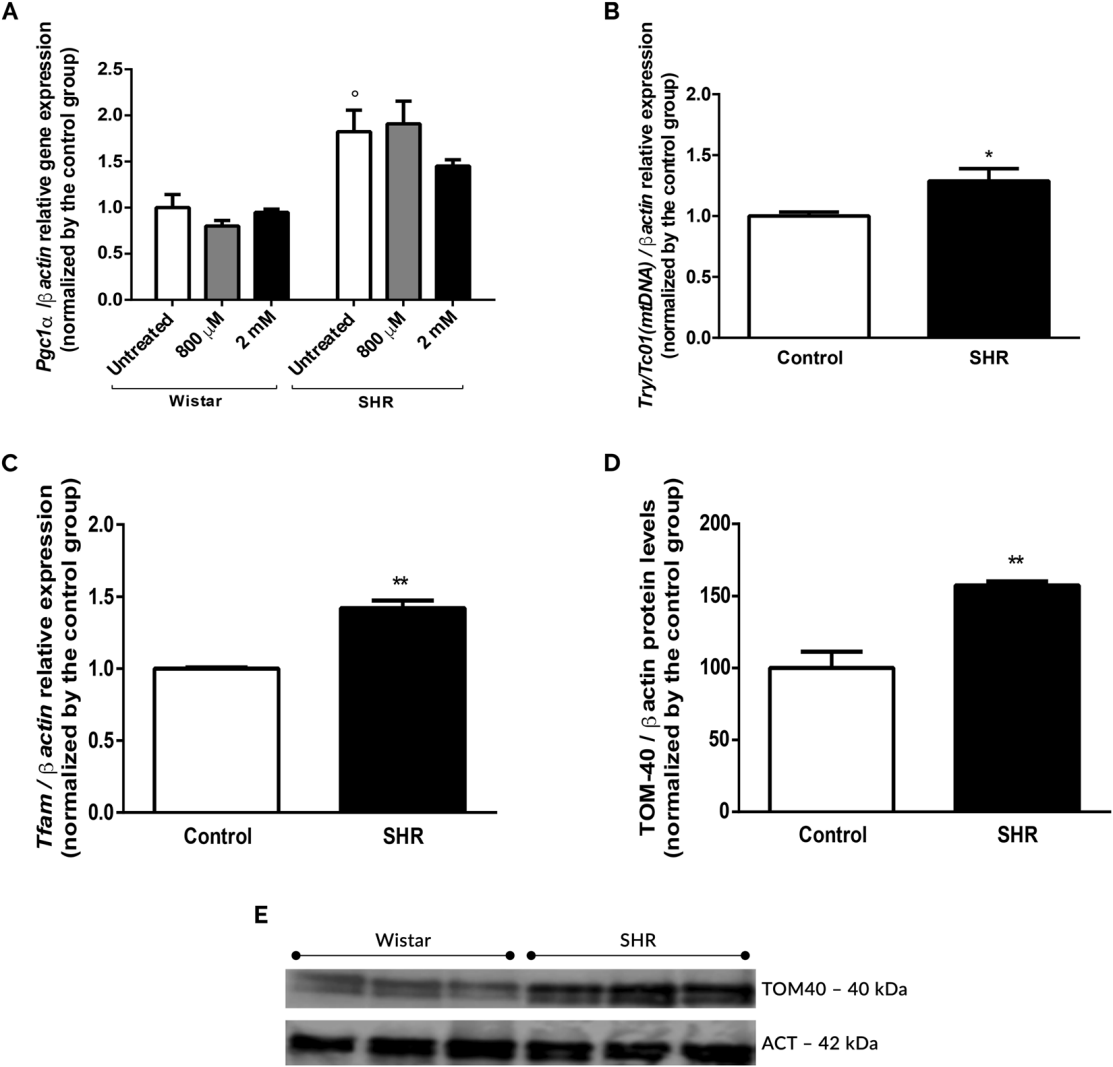
Increased mitochondrial biogenesis and mitochondria copy number in SHR astrocytes. Relative expression of *Pgc-1α* (**A**), *Mtco1* (mtDNA) (**B**) and *Tfam* (**C**), and TOM-40 levels (**D**,**E**) in astrocytes from Wistar and SHR animals in the presence and absence of CoCl_2_ treatment (800 µM and 2 mM, for 24 hrs). Data in graphs are the mean ± SD and the results were normalized as percentage of control group (N = 4, in duplicates). (**E**) is a cropped image; the uncropped blot is included in Supplementary Information. Statistical analysis was performed using Two-Way ANOVA followed by *post-hoc* Duncan or Student’s t Test. It was considered significant, p < 0.05; °p < 0.01, *p < 0.01 and **p < 0.001 in relation to Wistar group.

In order to evaluate changes in mitochondrial content, we assessed *Pgc1-α*, *Mtco1* and *Tfam* expression and TOM-40 levels^46–52^. As shown in Fig. 7A, there is a significant increase in *Pgc1-α* expression in astrocytes from SHR animals in relation to Wistar’s cells. Curiously, CoCl_2_ does not induce any significant changes (Fig. 7A). Corroborating this data, we demonstrated that SHR astrocytes show a significant increase in *Mtco1*^53–56^ (Fig. 7B) and *Tfam*^57–61^ expressions in SHR astrocytes (Fig. 7C). Concomitantly, we detect a significant increment in TOM-40 levels in SHR astrocytes (Fig. 7D,E). All these data reinforce that, indeed, an increased mitochondrial biogenesis as well as mitochondrial content in SHR astrocytes could be related to changes in mitochondrial metabolism.

### Intrauterine and chemical hypoxias lead to a decreased energy metabolism

**Figure 8:**
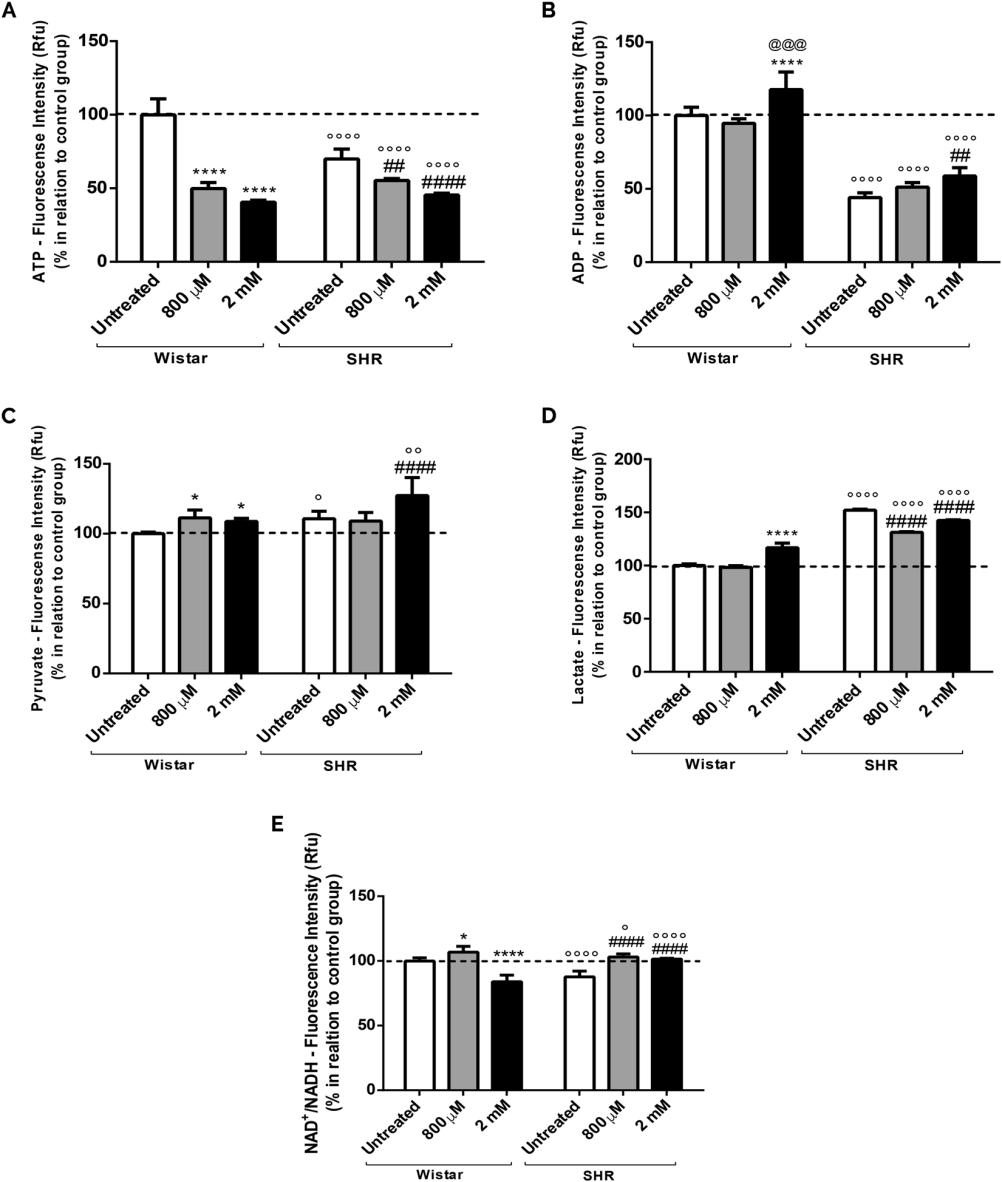
Alterations in high-energy compounds (ATP, ADP, Pyruvate, Lactate, NAD^+^ and NADH) after intrauterine and chemical hypoxia. Astrocytes were treated with CoCl_2_ (800 µM and 2 mM) for 24 hrs. (**A**) Measurement of ATP; (**B**) ADP levels; (**C**) Pyruvate analysis; (**D**) Evaluation of Lactate; (**E**) NAD^+^/NADH ratio. Data in graphs are the mean ± SD and the results were normalized as percentage of control group (N = 3, in duplicates). Statistical analysis was performed using Two-Way ANOVA followed by *post-hoc* Duncan and it was considered significant p < 0.05; ****p < 0.00001 and *p < 0.01, in relation to untreated Wistar group; ^@@@^p < 0.001, in relation to Wistar 800 µM group; ^####^p < 0.00001 and ^##^p < 0.001, in relation to untreated SHR group; °p < 0.01 and °°°°p < 0.00001, in relation to the respective Wistar group.

Since we demonstrated that astrocytes submitted to hypoxia present changes in calcium handling, mitochondria membrane potential and redox homeostasis, in addition to oxygen consumption (See Supplementary Fig. 2SS) and lipid peroxidation (See Supplementary Fig. 1SS), we investigated whether the increase in mitochondrial biogenesis and content could represent a compensation to keep cellular energy metabolism. As presented in Fig. 8A, there is a decrease in the ATP production in SHR astrocytes when compared to the untreated Wistar group (Fig. 8A). On top of that, when cells are exposed to chemical hypoxia, ATP levels are drastically reduced (Fig. 8A). Regarding to ADP levels, we note a reduction in SHR astrocytes in relation to the untreated Wistar group. On the other hand, after intense chemical hypoxia, there is a significant augmentation in ADP (Fig. 8B).

In order to verify if changes in ATP and ADP are accompanied by changes in intermediate metabolic products, Pyruvate and Lactate levels were measured. Figure 8C show an increase in Pyruvate on SHR astrocytes (untreated) in relation to Wistar cells. Curiously, chemical hypoxia induces a significant enrichment in Pyruvate in both Wistar and SHR astrocytes (Fig. 8C), indicating that either SHR astrocytes can have an increase in glycolytic pathway, or Pyruvate is being less converted into acetyl-CoA.

As an increase in Pyruvate may be favoring Lactate production, and hypoxia can induce an increase in lactate dehydrogenase A activity^35,62,63^, we also analyzed Lactate levels. We observe that there is a significant increase in Lactate production in SHR astrocytes when compared to Wistar untreated cells (Fig. 8D). Interestingly, only high-intensity chemical hypoxia was able to promote an augmentation in Lactate production in Wistar astrocytes.

To evaluate whether hypoxia is influencing the aerobic pathways through changes in high-energy compounds, we evaluated the levels of NAD^+^ and NADH. There is a significant reduction in the ratio NAD^+^/NADH in SHR cells in comparison to Wistar’s; the same result is obtained in Wistar astrocytes exposed to intense chemical hypoxia. On the other hand, moderate hypoxia on Wistar astrocytes, as well as moderate and intense hypoxia on SHR astrocytes, lead to a significant increment in the NAD^+^/NADH (Fig. 8E).

The compilation of all these data show that there is an energetic disturbance in SHR, despite increased biogenesis, and a worsening of the situation is noticed after exposure to intense hypoxia. Interestingly, the same occurs after induction of chemical hypoxia in Wistar astrocytes.

## Discussion

Along this manuscript, we showed that hypoxia (intrauterine, chemical and gaseous) could induce mitochondrial deregulation and consequently diminishment in energy metabolism in primary astrocytes. Curiously, astrocytes submitted to intrauterine hypoxia only, seem to activate compensatory mechanisms, namely mitochondrial biogenesis. Since SHR are described in literature as a SZ model, we further speculate that our data can be likewise related to phenotypic alterations and neuropathological features observed in these animals.

In our systems, we firstly investigated if CoCl_2_ treatment would be capable of hypoxia induction; thus we evaluated *Hif1α* expression^35,64–69^. Surprisingly, we observed a significant reduction in *Hif1α* (Fig. 2), that could be related to the presence of atmospheric oxygen leading to *Hif1α* degradation^70,71^. Indeed, reports mention that this degradation is fast^70^ and only *Hif1α* would not be sufficient to indicate hypoxia activation. Therefore, we evaluated the vascular endothelial growth factor (*Vegf*), which is the most abundant secondary element after the accumulation and activation of *Hif1α*^37–39^. We observed a significant increase in *Vegf* expression after intrauterine and chemical hypoxias, demonstrating that SHR and CoCl_2_ stimulation are capable of hypoxia activation.

To understand the processes in which hypoxia could be able to cause abnormalities in mitochondrial function, we used several methods to evaluate mitochondrial metabolism, including the evaluation of Ca^2+^ homeostasis (Fig. 3). Concerning to that, our data show that SHR astrocytes, in comparison to Wistar’s, presented less Ca^2+^ in the cytosol, but more Ca^2+^ were release from mitochondria after FCCP. Interestingly, it was described that periods of intrauterine hypoxia have the ability to modify Ca^2+^ levels causing ion accumulation in the cytosol and bringing damage to the developing fetus^72^. Concomitantly, chemical and gaseous hypoxias also induced an increase in mitochondrial Ca^2+^ uptake (Fig. 3). Such process would act as a protective factor avoiding accumulation of Ca^2+^ in the cytosol and the activation of cell death^72,73^. Consequently, the intrauterine (SHR), chemical and gaseous hypoxias could be inducing an increase in mitochondrial Ca^2+^ uptake as a protective mechanism. This data was not yet observed, and it is being reported herein for the first time.

Since an increase in mitochondrial Ca^2+^ can also be detrimental, since leads to a deregulation of mitochondrial membrane potential^58^ favouring the exit of cytochrome C^74–77^, we further evaluated mitochondrial membrane potential (∆Ψ_m_) in our cellular models. We observed that all kind of hypoxias tested herein induced changes in mitochondrial polarization (Fig. 4). Therefore, the higher retention of Ca^2+^ in the mitochondrial matrix can be due its membrane potential^74–76^. Curiously, we detected, after FCCP, an increase in TMRE cytosolic fluorescence in SHR cells in relation to untreated Wistar astrocytes, even though these cells present more TMRE in the cytosol (Fig. 4). This result seems contradictory, although it can imply that SHR astrocytes have more mitochondria. Consequently, the increased release of TMRE after FCCP addition could be a reflection of mitochondria augmentation^40,41,47,48^. Specifically after CoCl_2,_ there is a significant decrease in TMRE fluorescent signal after FCCP, indicating that organelles are depolarized; similar results were obtained after cells were exposed to 0% of O_2_ (Fig. 4). Conversely, this result was not obtained in SHR astrocytes after 8 hours of hypoxia. This event may suggest that short periods of gaseous hypoxia could favour polarization of mitochondria instead. Turcotte *et al*. using cells resistant to hypoxia demonstrated that this cell type suffers less from changes caused by hypoxia and tends to ensure ∆Ψ_m_ modifications^78^. Thus, one can suggest that SHR astrocytes could be more tolerant to gaseous hypoxia because they have already experienced an intrauterine lack of O_2_ as a preconditioning status.

To a better understanding of mitochondrial function after hypoxia, we also evaluated oxidative stress in astrocytes. As we can observe in Fig. 5, intrauterine hypoxia and CoCl_2_ treatment on Wistar astrocytes induced a significant increment in the DCF signal; chemical hypoxia did not interfere in SHR ROS production. In fact, some studies have proposed that SHR strain is inclined to have genetically based modifications in the redox system, which seems to be correlated with long periods of intrauterine hypoxia^79^. Curiously, gaseous hypoxia in SHR astrocytes decreased DCF signal. In this case, it is hypothesized that a severe hypoxia could modify the redox homeostasis leading the cell to a metabolically collapse, decreasing the DCF signal^80–82^. Alternatively, it can induce an increase in antioxidant capacity, which would decrease ROS. In fact, it was previously described that there is augmentation in antioxidant enzymes (catalase, GSH and SOD) in varied cell types exposed to hypoxia^83–85^.

Because DCF is capable of providing data about general ROS synthesis, we sequentially evaluated Mitosox fluorescence (in order to check O_2_^°−^ levels) (Fig. 5). Equally, astrocytes from SHR presented a significant intensification in O_2_^°−^ production. In addition, we detected an increase in O_2_^°−^ in Wistar and SHR astrocytes exposed to intense chemical hypoxia. However, after gaseous hypoxia, we only observed an increase in Mitosox fluorescence in Wistar cells after 18 and 24 hours, and after 18 hours in SHR groups. The drastic reduction of Mitosox after 24 hours of gaseous hypoxia, can be due to modifications in redox metabolism after long periods of hypoxia (intrauterine)^81,82^ or, alternatively, to a mitochondrial destabilization and even metabolic collapse, that could in part, explain the less superoxide synthesis^82,83^.

To check if intrauterine hypoxia could also lead to an increase in lipid peroxidation, which would be expected since we demonstrated an enrichment in DCF and Mitosox signals, we evaluated malondialdehyde (MDA) and 4-hydroxynonenal (HNE) levels (supplementary material)^86–89^ (See Supplementary Fig. 1SS). We revealed that SHR astrocytes presented a significant increment in MDA and 4-HNE levels in comparison to control cells. Hence, SHR astrocytes could be, in fact, particularly susceptible to changes in mitochondrial function and metabolism because they are continuously exposed to modification of redox status^79,81^.

Taking redox homeostasis data altogether, we latter checked changes in *Nef2l2* and *Nrf1* expression, genes directly implicated in cellular detoxifying systems^42–45,90^ and in the regulation and transcription of several nuclear-encoded electron transport chain proteins^45^, respectively. Interestingly, no changes were detected between Wistar and SHR groups regarding *Nef2l2* and *Nrf1* (Fig. 6). Though, there was an increase in *Nef2l2* expression in Wistar cells treated with 800 µM CoCl_2_ (moderate hypoxia), but not in SHR. Interestingly, it was already demonstrated that short periods of hypoxia could favour the antioxidant response^64,81^. Looking to the groups exposed to intense hypoxia, we detected a drastic reduction in both *Nef2l2* and *Nrf1* expression in Wistar and SHR astrocytes (Fig. 6). Remarkably, increased levels of ROS have been related to the fall in *Nfe2l2* levels in some cell types, such as epithelial and cardiac^42–44^ in the sense that during intense oxidative stress, there is a failure in the translocation of the gene to its binding site in the cell nucleus, reducing the transcription of secondary factors of the antioxidant cascade^44^.

Recalling mitochondrial deregulation, we postulated that perhaps intrauterine hypoxia could increase mitochondrial content and biogenesis in astrocytes to compensate changes in calcium handling, mitochondrial potential and redox homeostasis. Corroborating our hypothesis, we showed that SHR astrocytes presented a significant increase in *Pgc1-α* expression, the central regulator of mitochondrial biogenesis^46,47^ (Fig. 7), indicating that SHR strain would be predisposed to mitochondrial biogenesis. Concordantly, there was a significant increase in mitochondrial encoded complex IV subunit cytochrome c oxidase I (*Mtco1*)^53,56,91^ expression, a candidate gene downstream of *Pgc-1α*, and in *Tfam* expression, a transcription factor that activates transcription of mtDNA^61^ and is involved with mitochondrial formation and replication^57–61^. Corroborating our findings, we also showed a significant increase in TOM-40 level, a mitochondrial translocase of the outer membrane used for estimate the amount of mitochondrial in different cell types^91–93^ (Fig. 7). Interestingly, it was already demonstrated alterations in mtDNA copy number in SZ too^94–96^ as an association between mtDNA mutation and cognitive deficits in SZ’s subjects^97^. Since the hypoxia-effect on mitochondrial biogenesis was never described previously in SHR astrocytes, our data is the first that suggest that SHR cells could compensate mitochondrial dysfunction induced by intrauterine hypoxia by increasing mitochondrial content.

Due to all these findings, we also evaluated oxygen consumption in our cellular models (See Supplementary Fig. 2SS). The analysis revealed that there was no change in O_2_ consumption between untreated Wistar and SHR astrocytes. This result indicates that SHR astrocytes need more mitochondria to keep the same rate of oxygen consumption. Nonetheless, we saw a decrease in respiration in Wistar cells exposed to moderate chemical hypoxia and an increase in the O_2_ consumption in the astrocytes from both groups exposed to intense chemical hypoxia. Thereupon, we suggest that the elevated respiration rate, along with higher O_2_^°−^ concentration, can be, in fact, related to uncoupled mitochondria. Consequently, the organelle would consume more O_2_ pursuing to keep cellular energy metabolism^98,99^.

Afterwards, we evaluated the metabolites responsible for the regulation of cell’s energy pathways, named ATP, ADP, Pyruvate, Lactate, NAD^+^ and NADH levels. As shown in Fig. 8, there was a reduction in ATP levels in SHR cells in relation to controls. Interestingly, chemical hypoxia induces a further decrease in ATP in both Wistar and SHR astrocytes. It is worthy to mention that in the context of SZ, changes in mitochondrial energetic metabolism, including a decrease in ATP, was already described^100–103^. When we observed ADP, we also detected a decrease in SHR cells in relation to Wistar’s. Despite of that, intense chemical hypoxia led to higher ADP in both groups. This outcome can suggest that these cells can be consuming the available ATP to modify energetic pathways, such as the blockage of Pyruvate Dehydrogenase activity^64^. Besides, it is known that hypoxia induces inactivation of pyruvate dehydrogenase and a further reduction in the acetyl-coa supply, decreasing the activity of tricarboxylic acid cycle (TCA) and oxidative phosphorylation^35,62,64^. It was also demonstrated that hypoxia increases the activity of enzymes from glycolytic pathway in attempt to keep energetic requirement^35,62^. Consequently, Pyruvate levels would increase. Indeed, we saw a significant increase in Pyruvate in astrocytes exposed either to intrauterine or chemical hypoxia. Thus, the reduction of ATP and the increase in ADP can be related to the increase of Pyruvate induced by hypoxia^35,62,63^.

Moreover, it was already demonstrated that hypoxia induces an increase in the activity of Lactate Dehydrogenase A^62^ favouring Lactate synthesis. Concerning to that, our data shows a significant increase in Lactate levels in untreated SHR cells in relation to Wistar astrocytes (Fig. 8). Specifically, in Wistar group, only intense hypoxia was able to cause this effect. Interestingly, a cohort study with SZ’s persons presented a correlation between negative symptoms, increase in lactate levels and function of mitochondrial complex III^104^. Moreover, it was recently described that there is an increase in lactate levels in SZ’s subjects’ brain, in DISC1 (disrupted in schizophrenia 1) mouse model, as well as in inducible pluripotent stem cells (iPSCs) from a schizophrenia individuals^105^. As well, our results demonstrate that SHR astrocytes submitted to CoCl_2_ treatment present a decrease in Lactate in relation to untreated SHR cells, indicating that Lactate could be reappointed to other intracellular signalling pathways. It is known that under intense hypoxia a great amount of Lactate is extruded or deviated to glutamate synthesis^106–108^. In this case, hypoxia can promote the activation of monocarboxylate transporter 4 (MCT4), which transports the Lactate out of the cell^106^. Furthermore, it can be suggested that higher levels of Lactate could provide more substrates for the conversion of glutamine to glutamate and could increase glutamate oxidation and its conversion into alpha-ketoglutarate; both processes are recognized as the essential functions to astrocytes^106–108^.

We further demonstrated that after intrauterine and intense chemical hypoxia in Wistar cells there was a significant decrease in the NAD^+^/NADH ratio, result that can be due to an increased reconversion of Lactate into Pyruvate (Fig. 8). Additionally, knowing that mitochondria under hypoxia is dysfunctional, the lower formation of NAD^+^ and a consequent increase in NADH can be due to the incapability of its reconversion^109,110^. On the contrary, SHR astrocytes submitted to intense chemical hypoxia, presented an augmentation of NAD^+^/NADH ratio. In fact, the decrease in NADH could be an attempt of Pyruvate formation, from Lactate, to achieve a higher production of ATP^108^. Moreover, this can reflect an increased activity of the respiratory chain that, in turn, consume more NADH (decreasing its levels) and produce more NAD^+^^109,110^. Because there is no ATP production under this situation, even after mitochondrial biogenesis, it is possible that the organelles are uncoupled.

Altogether, this work brings new insights into how hypoxia is connected to mitochondrial deregulation, and how intrauterine hypoxia could be related to neuronal dysfunction, since SHR is an animal model of SZ. Consequently, we do hope that our results can contribute to new findings regarding psychiatry disorders target therapy.

## Material and Methods

### Animal models

For the present study, our group used Wistar (control group) and SHR (Spontaneous Hypertensive Rats) pups. Colonies of both lines were maintained at the housing facility of Santa Casa de São Paulo School of Medical Sciences; the first breeding pairs were acquired from University of São Paulo (USP) (Veterinary Medicine Faculty), São Paulo, Brazil.

All adult animals were housed in number of five in plastic cages measuring 33 × 40 × 17 cm. They were kept in rooms with ventilation and temperature control system (20 to 23 °C) in light-dark cycle of 12/12 hours; light phase starts at 6:30 am. The mating method for both strains was “harem”; it means that for each male, two females were added in the cage. Water and food were provided *ad libitum*.

All the experimental procedures, including euthanasia and decapitation, were performed according to the ethical principles for the use of laboratory animals described by the Brazilian Society for Laboratory Animal Science (SBCAL), National Council for Control of Animal Experimentation (CONCEA), Arouca Law (approved in 2008) and the Ethics Committee on Animal Use of Santa Casa de São Paulo School of Medical Sciences (CEUA Santa Casa). Our experimental protocols were carried out in accordance with the guidelines and they were approved by the number 003/15 by our Institutional Committee (President of CEUA Santa Casa, Dra. Fernanda Vasquez Daud).

### Spontaneously hypertensive rat (SHR)

SHR is a genetically hypertensive animal characterized by an increase in the arterial tension^111^ and, as a result, SHR females impose an intrauterine hypoxia situation to their embryos^112,113^. Curiously, several phenotypic hallmarks observed in newborn rats are also shown in humans when they are exposed to intrauterine hypoxic conditions due to their hypertensive mothers, named growth retardation, neurodevelopmental impairment and cognitive deficits^112,113^. Concomitantly, SHR females present an abnormal expression of placental proteins and deregulation of the renin-angiotensin-aldosterone system, as described in hypertensive women^114,115^. Thus, it is agreed that SHR rats are often used to mimic gestational hypertension^112,116^.

Due to modifications in neural circuit, as well as behavioral phenotypes present in these rats, SHR started to be used to investigate psychiatric disorders such as SZ^117^. Indeed, SHRs present hyper locomotion (correlated with SZ positive symptoms), social interaction deficit (associated to SZ negative symptoms) and decreased contextual fear conditioning, which denotes cognitive symptoms and diminished emotional processing. In addition, SHR show increased rearing behaviour (associated with positive symptoms in human beings) and decreased performance in prepulse inhibition of startle (PPI) (a pre-attentional information processing test corresponding to positive/cognitive symptoms in humans)^115–124^. PPI model has been used to study sensorimotor deficits related to SZ^124,125^. Specifically, regarding the augmentation in rearing phenotype in social context, it is also considered an antisocial behaviour, indicating that the animal prefers to explore the environment to have to interact with an unfamiliar rodent^114,115^. Therefore, it can be interpreted as a negative symptom as well.

It is worth mentioning that all phenotypes presented by SHR were pharmacologically validated for SZ, since they were reversed with antipsychotics used in the clinic for patients, but not with psych stimulant drugs, mood stabilizers or anxiety modulators^114,123^. These findings show not only that SHR present a hypofunctional prefrontal cortex and a hyperfunctional nucleus accumbens, but also reinforce the intersections between contextual fear conditioning deficit and emotional processing abnormalities in SZ, strengthening the use of SHR as an animal model of this disorder.

Recently, an article to validate behavioral variables observed in the SHR strain that could act as a general index of schizophrenic behavior was published^126^. Interestingly, all the behavioural assessments were re-evaluated and tested for schizophrenia-like trait (SLT) indices (such as locomotor activity, social interaction, PPI and Fear Conditioning test). It was found that these behavioural parameters revealed a SLT as a *continuum*, a result according to the nature of SZ in humans^126^. A matter of great importance is the fact that a deficit in emotional memory of SHR^114,117,118^ is reverted by antipsychotics^114^. Interestingly, these drugs are also used in SZ therapy, suggesting that the SHR strain could be used as a model for SZ-associated emotional memory deficits^117^. On the contrary, and in agreement with SHR as a SZ-model, SHR animals treated with amphetamine present an exacerbation of overall symptoms linked to SZ^114^. Thus, experimental evidences reinforce the behavioral profile of SHR associated with SZ. Neurochemical evidences also suggest that SHR present SZ-related features^127^. Actually, they present amygdala activation and prefrontal cortex deficits^126,128–131^, in addition to an increase in the release of dopamine induced by amphetamine into striatum^130^. Similarly, there is a decrease in the expression of dopaminergic receptors of type D4 (belonging to the family of D2 receptors) in the cortex of SZ carriers and SHR rats^131^, and an increase in dopamine transporters (DAT) in striatum of SHR^131–134^ (more information on Supplementary Material).

### Primary culture of astrocytes

In order to perform primary culture of astrocytes, we used cortexes from 3–4 days-old pups^135,136^. Animals were sacrificed and their brains were removed and transferred to a petri dish containing Dulbecco’s Modified Eagle Medium (DMEM). The cortex was dissected and dispersed mechanically with scissors and, subsequently, we used three-gauge needles (24G × ¾″, 26G × ½″ and 30G × ½″) for complete tissue dissociation. After this procedure, cell suspension was centrifuged at 435 g for 5 minutes (centrifuge 5810R − Eppendorf) and the supernatant was discarded. The final pellet was resuspended in 2 mL of DMEM supplemented medium (10% of fetal bovine serum and 1% of antimycotic/antibiotic) and the cells were distributed in T75 cm² flasks. The culture was maintained at 5% CO_2_ and 37 °C, and the medium was changed every four days. To each T75 cm² culture flask, it was used cortexes from three brain hemispheres. Thus, three animals (six hemispheres of cortex) were enough to two T75 cm² flasks. Each *N* sample was considered per litter, and not for each newborn. We did not consider sex or weight of the newborn animals.

### Cellular model of chemical hypoxia (CoCl_2_)

To mimic cellular hypoxia, primary astrocytes were treated with cobalt chloride (CoCl_2_). CoCl_2_ is known as a chemical inducer of hypoxia since it blocks the degradation of the transcription factor Hypoxia Inducible Factor 1 alpha (*Hif1α*) leading, consequently, to the increased activation of the hypoxia cascade. In normoxia situation (normal concentrations of O_2_), there is the hydroxylation of HIF1α by prolyl-hydroxylases (PHDs), which in turns, leads to its degradation through the ubiquitin proteasome system (UPS)^35,64,65^.

In our experiments, CoCl_2_ was dissolved in water and latter, following its physicochemical standards, it was aliquoted at 50 mM stock solution. We used along this study 800 µM, 1 mM, 2 mM and 5 mM of CoCl_2_ for 24 hours (hrs). In order to mimic a moderate and intense hypoxia, we used 800 µM and 2 mM of CoCl_2_, respectively.

### Cellular model of gaseous hypoxia (Hypoxia Chamber – 0% O_2_)

To ascertain the action of the hypoxia cascade on mitochondrial metabolism without the use of a chemical inducer, we also used the hypoxia chamber as a strategy (Hypoxia chamber Stemcell Technologies; Cat. No. 27310). Knowing that astrocytes are resistant to hypoxia^137,138^ and that 4%, 3% or even 1% of O_2_ over short periods, such as 8, 10 and 12 hours, did not alter cellular viability^137–139^, the primary culture from our lab was submitted to a regimen of 0% of O_2_ for 8, 18 and 24 hours. To establish the 0% of O_2_, we used a protocol based on the internal volume of the chamber (cm^3^), in which O_2_ are displaced by N_2_ in a volume of 30 L/min for 4 minutes^140^. In our experiments, to resemble moderate and intense gaseous hypoxia, we exposed the cells to 0% of O_2_ for 8 h, or 18 and 24 hours, respectively.

### Viability assay - chemical and gaseous hypoxias

Cell viability was assessed by the [3- (4,5-dimethylthiazol-2yl)-2,5-diphenyl tetrazolyum] bromide assay (MTT). Cells were plated in a 96-well plates (3 × 10^4^ cells per well) in duplicate, and after 24 hours of CoCl_2_ treatment or upon exposure to the hypoxia chamber at the proposed times, cells were washed with PBS 1× (PBS, mM: 0,14 NaCl, 2,7 KCl, 1,5 K_2_HPO_4_ e 8,1 Na_2_HPO_4_), and incubated with the MTT reagent at the final concentration of 12 mM at 37 °C for 4 hours (Vybrant ™ MTT Cell Proliferation Assay Kit, Molecular Probes). Following the manufacturer’s protocol, the 96-well plate was then allocated into a plate reader (spectrophotometer) to read the absorbance at 540 mm at 37 °C. All assays were performed with control cells as well (without any treatment and in normoxia). Cellular viability was normalized as percentage of control group (mean ± SD).

### Mitochondrial metabolism analysis – functional parameters

In order to evaluate Ca^2+^ levels and mitochondrial membrane potential (ΔΨ_m_), control and SHR astrocytes were re-plated in 96-well plates (6 × 10^4^ cells per well) for 24 hours as described previously^135,136,141,142^. After this period, the cells were treated with the proposed concentrations of CoCl_2_ (800 µM or 2 mM) for 24 hours or exposed to hypoxia chamber (8, 18 or 24 hours). Specifically, to evaluate cytosolic Ca^2+^ level, cells were loaded with Fluo-4-AM (10 µM) plus Pluronic F-127™ (20%) (1 hour, 37 °C) (485 nm excitation; 525 nm emission). Mitochondrial membrane potential was accessed after incubating the cells with Tetramethylrhodamine ethyl ester (TMRE, 500 nM) (1 hour, 37 °C) (540 nm excitation; 590 nm emission). Both dyes were incubated in microscopy medium (mM: 120 NaCl, 3,5 KCl, 0,4 KH_2_P O_4_, 5 NaHCO_3_, 1,2 NaSO_4_, 20 HEPES, 15 Glucose e pH 7.4), supplemented with 1 mM of calcium chloride (CaCl_2_).

For the experiments related to redox homeostasis, cells were also re-plated in 96-well plates (6 × 10^4^ cells per well), but they were loaded in microscopy medium or Krebs medium (mM: 132 NaCl, 4 KCl, 1 CaCl_2_, 1.2 NaH_2_PO_4_, 1.4 MgCl_2_, 6 Glucose, 10 HEPES) supplemented with 1 mM of CaCl_2_ accordingly to the fluorescent probe. To evaluate general ROS measurements, cells were incubated in microscopy medium with Fluorescein Diclohidro Carboxylated Reagent (CM-H_2_DCF-DA) (20 µM; 30 min, 37 °C) (495 nm excitation; 520 nm emission)^135,136,141–143^; to assess superoxide (O_2_^°−^) levels, cells were loaded with Mitosox in Krebs medium (5 µM, 10 minutes at 37 °C) (510 nm excitation; 580 nm emission)^144^. The CM-H_2_DCF-DA shows much better retention in live cells tan DCFH_2_-DA and it is used to detect the generation of reactive oxygen intermediates. CM-H_2_DCFDA passively diffuses into cells, where the acetate groups are cleaved by intracellular esterases becoming DCFH, which is retained inside the cell. Afterwards, it is rapidly oxidized to the highly green fluorescent compound 2,7-dichlorofluorescein (DCF)^141–143^. On the other hand, Mitosox is a cationic derivative of dihydroethidum designed for highly selective detection of superoxide in the mitochondria of live cells. The cationic triphenylphosphonium substituent of MitoSOX Red indicator is responsible for the electrophoretically driven uptake of the probe in actively respiring mitochondria^144^. Once in the mitochondrial matrix, the radical is oxidized by superoxide and the radical become fluorescent.

The Fluo-4-AM, TMRE and DCF fluorescence were acquired on a spectrofluorimeter (Spectramaxi3™) for a period of 5 minutes, to get basal signal, and for another 5 minutes after the addition of fluorocarbonyl cyanide phenylhydrazone (FCCP) (5 μM), a mitochondrial protonophore used as an internal experimental control to obtain maximum fluorescence and the delta value (Δ). The baseline fluorescence was calculated from the mean of the last 10 reading points before FCCP, while the delta (Δ) was calculated by the difference between the maximum fluorescence after FCCP and the basal fluorescence intensity^135,136,141–143^. All these experiments were performed in duplicate and data were expressed as percentage of control cells (mean ± SD).

Regarding Mitosox, a kinetic reading was performed for 1 hour and 30 minutes on the spectrofluorimeter. The results were obtained as RFU (relative fluorescence units) per minute, and data was expressed by the slope of the curve^144^. After reading, the medium was removed, cells were washed with PBS 1x and then they were incubated with 0.2% TRITON (for 30 minutes on ice). This process is required for protein extraction in order to normalize the slope per mg of protein^144^. Proteins were quantified by the Bradford method using the Bradford Quick Start reagent (BioRad) and data were expressed as percentage of control cells (mean ± SD).

### Mitochondrial function and hypoxia cascade activation – Gene expression

The expression of transcription factors related to mitochondrial metabolism was evaluated by real-time PCR (qPCR)^145,146^. For each reaction, performed by Universal SYBR®Green Supermix, it was used 200 ng of cDNA and sequence-specific primers (300 nM); the analysis of mitochondrial content was also evaluated through qPCR. The template was performed with an initial cycle of 30 secs at 95 °C, followed by 46 cycles of 15 secs at 95 °C plus 30 secs at 61 °C. After amplification, melting temperature of the PCR products were determined by performing melting curves, with 0.5 °C increments every 5 secs, from 65 °C to 95 °C. Primers sequences were as follow: I) *Peroxisome proliferator-activated receptor gamma coativator 1-alpha* (α) (*Pgc1-α*) (Forward 5′-3′: CTCGACACGGAGAGTTAAAGG, Reverse 5′-3′: TAAACTGAGCTACCCTTGGG); II) *Mitochondrial Transcription Factor A* (*Tfam*) (Forward 5′-3′: CAGAGTTGTCATTGGGATTGG, Reverse 5′-3′: CATTCAGTGGGCAGAAGTCC); III) *Nuclear Respiratory Factor 1* (*Nrf-1*) (Forward 5′-3′: GCTAAGGCTGCTGGGAAGTA, Reverse 5′-3′: TCAGTTGCTGTGGCGAGTTA); IV) *Nuclear Factor (erythroid-derived 2) Like 2* (*Nfe2l2*) (Forward 5′-3′: CTGCTGCCATTAGTCAGTCG, Reverse 5′-3′: GTGCCTTCAGTGTGCTTCTG); V) *Mitochondrial-encoded Complex IV Subunit Cytochrome c Oxidase Subunit 1* (*Mtco1*) (Forward 5′-3′: TGGCTTCGTCCACTGATTCC, Reverse 5′-3′: CGAGGTATCCCCGCTAATCC); VI) *β-actin*, that was used as housekeeping gene (Forward 5′-3′: AGGGAAATCGTGCGGTGAC, Reverse 5′-3′: CGCTCATTGCCGATAGTG).

At the end of each cycle, SYBR®Green fluorescence was recorded to enable determination of Cq. After amplification, melting temperature of the PCR products were determined. For qPCR analysis, the BioRad CFX Manager software was used. Normalization was carried out in relation to *β-actin* expression and data were expressed as percentage of control cells (mean ± SD).

### Mitochondrial content investigation – protein levels

To evaluate protein level related to mitochondrial content, total fraction from astrocytes culture were obtained as described previously^141,144–147^. In brief, extracts were prepared in ice-cold RIPA buffer (mM: 150 NaCl, 50 Tris HCl – pH 7.4, 5 EGTA; still containing 1% TRITON, 0.5% DOC, 0.1% SDS) (pH 7.5) supplemented with MS-SAFE protease and phosphatase inhibitor (Sigma-Aldrich) accordingly to manufacturer’s protocol. The final supernatant was collected and stored at −80 °C for later use. Samples were prepared using 50 μg of protein. Proteins were transferred to PVDF membrane (0.2 µM) and were incubated overnight, at 4 °C, with primary antibodies directed against: I) TOM-40 (Santa Cruz – SC-11414; 1:1000), protein coupled to mitochondrial outer membrane and II) ACTIN (Sigma – A2103; 1:5000). The secondary antibodies were Horseradish Peroxidase (HRP) pAb goat anti-mouse IgG, HRP pAb goat anti-rabbit IgG and IgG rabbit anti-goat-conjugated peroxidase. Immunoreactive bands were visualized with ECL substrate (GE - Healthcare) in ImageQuanty LAS 500 (GE-Healthcare). The Quantity One software (Biorad) was used to analyze the optical density of all bands. All data were expressed as percentage of control cells (mean ± SD) after normalization by ACTIN.

### Measurement of adenine nucleotides and metabolic compounds

To comprehend the relationship between mitochondrial dysfunctions and hypoxia, we further evaluated the levels of bio products related to energy metabolism, named ATP, ADP, Pyruvate, Lactate, NAD^+^ and NADH^141,145–147^. All of them were measured using commercially available kits (Abcam, ab83355, ab83359, ab65342, ab65330 and ab65348).

For the analysis, astrocytes (from Wistar and SHR pups) were plated at the concentration of 1 × 10^6^ cells/well (6-well-plate) and exposed with the proposed concentrations of CoCl_2_ (800 µM and 2 mM). After treatment, the procedure was started following information from the proposed kits (ab83355, ab83359, ab65342, ab65330 and ab65348). For all analysis, ATP, ADP, Pyruvate, Lactate, NAD^+^ and NADH levels were normalized for the number of cells (1 × 10^6^/N) and expressed as percentage of control cells (mean ± SD).

### Analysis of results and statistical analysis

Results are expressed as mean ± SD of the number of independent experiments indicated in figure legends. The graphs were assembled using the GraphPad Prism 6 program (GraphPad Prism Version 6.0), and the statistical analysis was performed with One- or Two-Way ANOVA followed by *post-hoc* test Duncan. Student’s t Test was performed when necessary. It was considered statistically different p < 0.05.

## Supplementary information

Supplementary Material

## Data Availability

All data generated or analysed during this study are included in this published article (and its Supplementary Information files).
