# Supplementary material for: Mitochondrial Dysfunction and Changes in High-Energy Compounds in Different Cellular Models Associated to Hypoxia: Implication to Schizophrenia
Source: Sci Rep. 2019 Dec 2;9:18049. doi: 10.1038/s41598-019-53605-4 (PMC6889309; doi:10.1038/s41598-019-53605-4)
Supplement: Supplementary file 1 — Supplementary Material [file 41598_2019_53605_MOESM1_ESM.pdf]

SUPPLEMENTARY MATERIAL

MITOCHONDRIAL DYSFUNCTION AND CHANGES IN HIGH-ENERGY COMPOUNDS IN DIFFERENT CELLULAR MODELS ASSOCIATED TO HYPOXIA: IMPLICATION TO SCHIZOPHRENIA

Luiz Felipe Souza e Silva[1], Mariana Dutra Brito[1], Jéssica Mayumi Camargo Yuzawa[1], Tatiana Rosado Rosenstock[1*]

[1]Santa Casa de São Paulo School of Medical Sciences, São Paulo, Brazil

*Corresponding author:
Tatiana Rosado Rosenstock
Santa Casa de São Paulo School of Medical Sciences
Rua Dr. Cesário Motta Jr., 61 - São Paulo - SP - CEP: 01221-020
Phone/Fax: 55-11-3367-7790 (9045)
tatiana.rosado@fcmsantacasasp.edu.br
tati.farm@gmail.com

1. Material and Methods

1. Spontaneusly hypertensive rats (SHR) and Schizophrenia

Today, numerous tasks are performed in rodents in order to identify changes in their behavioral that can be referred to human disorders; this definitely helps researchers to understand the pathophysiology of several diseases[1-5], including SZ[6]. In fact, in Spontaneously Hypertensive Rats (SHRs) were identified and characterized various phenotypic alterations resembling SZ. SHRs, when submitted to various investigations, present hyperlocomotion, social interaction deficit, decreased contextual fear conditioning and decreased performance in prepulse inhibition of startle (PPI)[7-16]. Specifically, regarding the augmentation in rearing phenotype in social context, it is also considered an antisocial behaviour, indicating that the animal prefers to explore the environment to have to interact with an unfamiliar rodent[7,17,18]. The augmentation of exploratory behaviour may still be a reflection of working memory deficits, a cognitive symptom observed in SZ[6]. Interestingly, the rise in rearing phenotype is correlated to dopamine release in the striatum[6,19], a biochemical characteristic also observed in patients with SZ and linked to positive symptoms[20].

Despite such evidences, there are still manuscripts in the literature that use SHR as a model of Attention Deficit Disorder (ADHD). However, it is important noting that such studies use young SHR animals as well as Wistar Kyoto (WKY) as control; the use of WKY animals is not indicated, since they present depressive-like behaviour[21]. In addition, several studies report that the phenotype of SHR do not respond to psych stimulant drugs, which are used to treat ADHD; they even make the picture worse[17,15,18]. All these data reinforce the SHR as model for SZ.

1.2. Lipid peroxidation investigation

To evaluated lipid peroxidation status in Wistar and SHR astrocytes, we measured two lipid peroxidation byproducts, malondialdehyde (MDA) and 4-hydroxynonenal (HNE)[21-23]. Lipid Peroxidation (MDA) assay fluorimetric kit (Ab118970) is based on the formation of MDA-TBA adduct a product of MDA and thiobarbituric acid reaction. This assay detects as low as 0.1 nmol of MDA levels per well and it is easily detected by fluorescence (Ex/Em= 532/553 nm). Results were expressed as percentage of control cells and data were previously normalized by the number of cells per well ($1x10^6$ / well).

On the other hand, to analyze HNE levels, we used the western blot technique and we evaluated the levels of alpha-HNE protein (68 kDa) (JAICA-MHN020P; 1:3.000)[23]. Data were normalized to ACTIN levels and results were expressed as percentage of control group.

1.3. Mitochondrial function - Oxygen consumption

In order to evaluate oxygen (O_2) consumption, astrocytes from both control (Wistar) and SHR animals were re-plated on 6-well plates ($1x10^6$ cells per well) for 24 hours, and after they were treated with $CoCl_2$ (800 µM and 2 mM) for an additional 24 hours. Subsequently, cells were trypsinized and resuspended in Krebs medium (nM:132 NaCl, 4 KCl, 1 $CaCl_2$, 1.2 NaH_2PO_4, 1.4 $MgCl_2$, 6 Glucose, 10 HEPES)[24-27]. Cells were then transferred to an O_2 electrode chamber (DW1, Clark type electrode, Hansatech Instruments, Norfolk, UK) with a constant temperature (37°C) coupled to an analogue/computer system, after calibration for gas phase (following manufacturer's protocol). Afterwards, we measured basal O_2 consumption and the respiration rate after the addition of FCCP (10 µM)[24-27]. The rate of O_2 consumption was acquired as nmol/mL/min. After readings, protein content was obtained for normalization (cells were lysed using 1 M NaOH) and we performed Bradford assay. O_2 consumption is shown as nmol/mL/min/mg of protein normalized to control group (in percentage)[24].

2. Results

2.1. Increased lipid peroxidation in astrocytes after neonatal hypoxia

As we observed alterations in mitochondrial metabolism in SHR astrocytes when compared to control group, we decided to further investigate lipid peroxidation in both groups (Figure 1SS). As a side note, lipid peroxidation refers to the oxidative degradation of lipids, in which free radicals take electrons from the lipids (generally from cell membranes), resulting in cellular damage and even cell death.

To verify lipid peroxidation in our system, we evaluated two peroxidation byproducts, malondialdehyde (MDA) and 4-hydroxynonenal (HNE). As we can observe on Figure 1SS, there is a significant increase in MDA (µM/ml) levels in SHR astrocytes in relation to Wistar cells (*$p<0.01$). In addition, our results also demonstrated a significant augmentation in αHNE levels in SHR astrocytes (*$p<0.01$) (Figure 1SS).

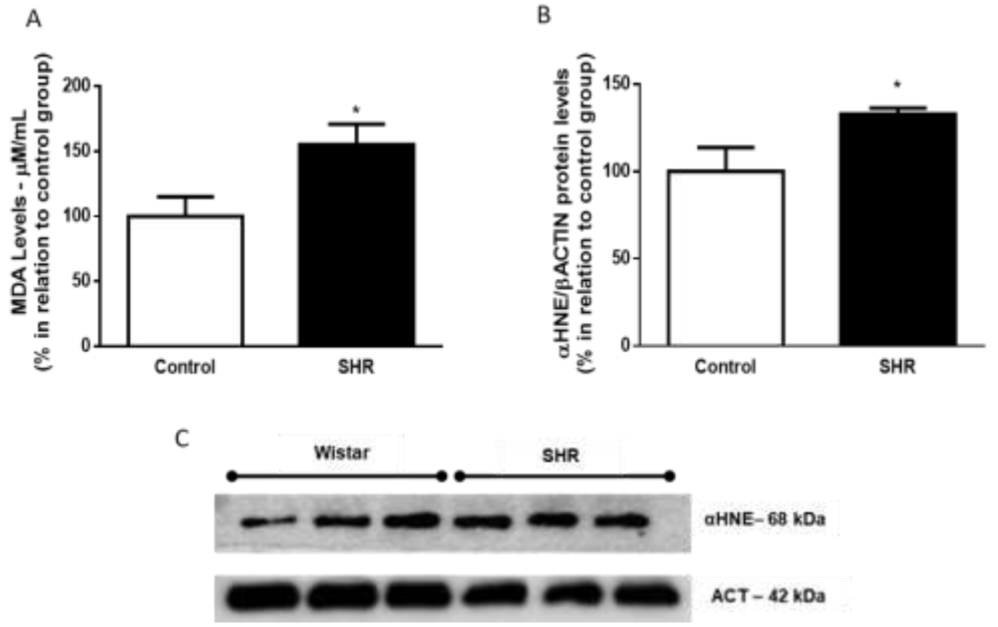

Figure 1SS: Increased lipid peroxidation in astrocytes from SHR cortex. Malonyldialdehyde levels measured through Lipid Peroxidation (MDA) Assay Kit (A) and α-4-hydroxynonenal (HNE) protein levels normalized to ACTIN (B and C). Data is represented by mean ± SD and the results were normalized as percentage of control group (N=3-4, in duplicates). Statistical analysis was performed using Student's t Test. It was considered significant $p<0.05$; $*p<0.01$, in relation to control group.

2.2. Increase in oxygen consumption after intense chemical hypoxia

The analysis revealed that there was no change in oxygen consumption between untreated Wistar and SHR astrocytes (Figure 2SS), indicating that despite changes in calcium levels, mitochondrial membrane potential and oxidative stress, the total respiration was not affected.

However, after chemical hypoxia, Wistar astrocytes showed a decrease in oxygen consumption in the presence of $CoCl_2$ 800 µM ($*p<0.01$) and an increase in the respiration after 2 mM of $CoCl_2$ treatment in relation to entreated cells (t* = $*p<0.01$) (Figure 2SS). Curiously, SHR astrocytes submitted to intense chemical hypoxia (2mM of $CoCl_2$) presented an augmentation of respiration rate of almost 200% in relation to untreated cells ($^{\#\#\#\#}p<0.0001$). When we compared between cell types (Wistar *versus* SHR) treated with $CoCl_{2,}$ we observed a significant intensification in O_2 consumption in SHR in comparison to the respective Wistar group (°°°°$p<0.0001$) (Figure 2SS).

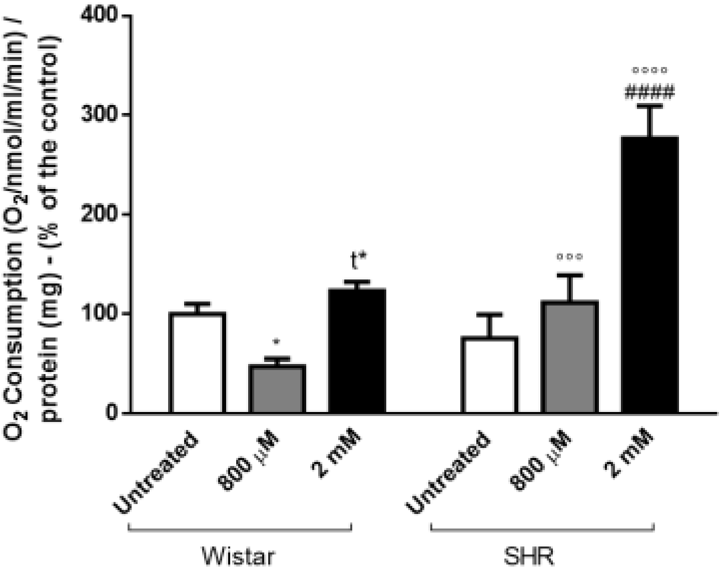

Figure 2SS: Increased O_2 consumption (nmol/mL/min/mg of protein) in astrocytes from Wistar and SHR astrocytes after intense chemical hypoxia. Cells were evaluated in the absence and presence of $CoCl_2$ (800 µM and 2 mM, 24 hrs). Data in graphs are the mean ± SD and the results were normalized as percentage of control group (N=3, in duplicates). Statistical analysis was performed using Two-Way ANOVA followed by *post-hoc* Duncan and Student's t Test. It was considered significant p<0.05; *p<0.01 and t*p<0.01, in relation to untreated Wistar group; $^{\#\#\#\#}$p<0.00001, in relation to untreated SHR group; °°°p<0.0001 and °°°°p<0.00001, in relation to the respective Wistar group.

2.3. TOM-40 levels augmentation in neonatal hypoxic astrocytes

As shown in Figure 7D and E, we detect a significant increment in TOM-40 levels in SHR astrocytes. All data presented reinforce that an increased mitochondrial content in SHR astrocytes could be related to changes in mitochondrial metabolism. The uncropped blots of ACTIN and TOM-40 are shown below (Figure 3SS and 4SS).

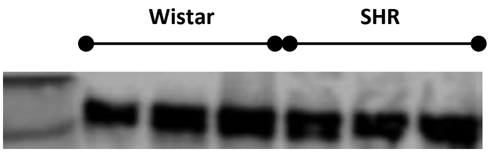

Figure 3SS: Uncropped blot of ACTIN levels in astrocytes from Wistar and SHR animals.

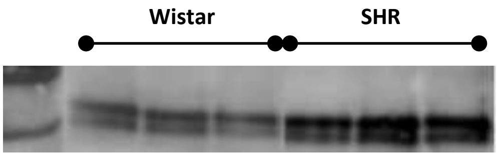

Figure 4SS: Uncropped blot of TOM-40 levels in astrocytes from Wistar and SHR animals.

3. References

1. Young, J. W., Zhou, X. & Geyer, M. A. Animal models of schizophrenia. Curr. Top. Behav. Neurosci. 4:391-343; 10.1007/7854_2010_62 (2011)

2. Lipska, B. K. & Weinberger, D. R. To model a psychiatric disorder in animals: Schizophrenia as a reality test. Neuropsychopharmacology 23(3):223-239; 10.1016/S0893-133X(00)00137-8 (2000).

3. Mattei, D. et al. Minocycline rescues decrease in neurogenesis, increase in microglia cytokines and deficits in sensorimotor gating in an animal model of schizophrenia. Brain. Behav. Immun. 38:175-184; 10.1016/j.bbi.2014.01.019 (2014).

4. Miyamoto, Y. & Nitta, A. Behavioral Phenotypes for Negative Symptoms in Animal Models of Schizophrenia. J. Pharmacol. Sci. 126(4): 310-320; 10.1254/jphs.14R02CR. (2014).

5. Ratajczak, P. et al. Influence of aripiprazole and olanzapine on behavioral dysfunctions of adolescent rats exposed to stress in perinatal period. Pharmacol. Reports. 65(1):30-43; 10.1016/S1734-1140(13)70961-7 (2013).

6. Lett, T. A., Voineskos, A. N., Kennedy, J. L., Levine, B. & Daskalakis, Z. J. Treating working memory deficits in schizophrenia: A review of the neurobiology. Biol. Psychiatry 75(5):361-370; 10.1016/j.biopsych.2013.07.026 (2014).

7. Mattace Raso, G. et al. Evaluation of Placental Protein Modifications in Normotensive and Spontaneously Hypertensive Rats. Placenta 29, 429–435; 10.1016/j.placenta.2008.02.002 (2008).

8. Pinto, Y. M., Paul, M. & Ganten, D. Lessons from rat models of hypertension: From Goldblatt to genetic engineering. Cardio. Res. 39, 77–88; 10.1016/s0008-6363(98)00077-7 (1998).

9. Calzavara, M. B. et al. Neuroleptic drugs revert the contextual fear conditioning deficit presented by spontaneously hypertensive rats: A potential animal model of emotional context processing in Schizophrenia? Schizophr. Bull. 35, 748–759; 10.1093/schbul/sbn006 (2009).

10. Ledoux, J. E., Sakaguchi, A. & Reis, D. J. Strain differences in fear between spontaneously hypertensive and normotensive rats. Brain Res. 277, 137–143; 10.1016/0006-8993(83)90915-0 (1983).

11. Palmer, A. A. et al. Prepulse startle deficit in the brown norway rat: A potential genetic model. Behav. Neurosci. 114, 374–388; 10.1037//0735-7044.114.2.374 (2000).

12. Ferguson, S. A. & Cada, A. M. A longitudinal study of short- and long-term activity levels in male and female spontaneously hypertensive, Wistar-Kyoto, and Sprague-Dawley rats. Behav. Neurosci. 117, 271–282; 10.1037/0735-7044.117.2.271 (2003).

13. Kinkead, B., Selz, K. A., Owens, M. J. & Mandell, A. J. Algorithmically designed peptides ameliorate behavioral defects in animal model of ADHD by an allosteric mechanism. J. Neurosci. Methods 151, 68–81; 10.1016/j.jneumeth.2005.07.015 (2006).

14. Vendruscolo, L. F. et al. A QTL on rat chromosome 7 modulates prepulse inhibition, a neuro-behavioral trait of ADHD, in a Lewis x SHR intercross. Behav. Brain Funct. 2; 10.1186/1744-9081-2-21 (2006).

15. Levin, R. et al. Spontaneously Hypertensive Rats (SHR) present deficits in prepulse inhibition of startle specifically reverted by clozapine. Prog. Neuro-Psychopharmacology Biol. Psychiatry 35, 1748–1752; 10.1016/j.pnpbp.2011.06.003 (2011).

16. Geyer, M. A., Krebs-Thomson, K., Braff, D. L. & Swerdlow, N. R. Pharmacological studies of prepulse inhibition models of sensorimotor gating deficits in schizophrenia: A decade in review. Psychopharmacology 156, 117–154; 10.1007/s002130100811 (2001).

17. Pinto, Y. M., Paul, M. & Ganten, D. Lessons from rat models of hypertension: From Goldblatt to genetic engineering. Cardio. Res. 39, 77–88, https://doi.org/10.1016/s0008-6363(98)00077-7 (1998).

18. Peres, F. F. et al. A schizophrenia-like behavioral trait in the SHR model: Applying confirmatory factor analysis as a new statistical tool. Prog. Neuro-Psychopharmacology Biol. Psychiatry. 85, 16–22; 10.1016/j.pnpbp.2018.03.021 (2018).

19. Thiel, C. M., Huston, J. P. & Schwarting, R. K. W. Cholinergic activation in frontal cortex and nucleus accumbens related to basic behavioral manipulations: Handling, and the role of post-handling experience. Brain Res. 812(1-2):121-132; 10.1016/S0006-8993(98)00961-5 (1998).

20. Tamminga, C. A. & Medoff, D. R. The biology of schizophrenia. Dialogues Clin. Neurosci. 2, 339–348 (2000).

21. Overstreet, D. H. Modeling depression in animal models. Methods Mol. Biol. 829:125-44; 10.1007/978-1-61779-458-2_7 (2012).

22. Andringa, K. K., Udoh, U. S., Landar, A. & Bailey, S. M. Proteomic analysis of 4-hydroxynonenal (4-HNE) modified proteins in liver mitochondria from chronic ethanol-fed rats. Redox Biol. 2:1038-47; 10.1016/j.redox.2014.09.006. (2014).

23. Gaschler, M. M. & Stockwell, B. R. Lipid peroxidation in cell death. Biochem. Biophsy. Res. Commun. 482(3):419-425; 10.1016/j.bbrc.2016.10.086 (2017).

24. Naia, L. et al. Activation of IGF-1 and Insulin Signaling Pathways Ameliorate Mitochondrial Function and Energy Metabolism in Huntington’s Disease Human Lymphoblasts. Mol. Neurobiol. 51(1):331-48; 10.1007/s12035-014-8735-4 (2014).

25. Rosenstock, T. R. et al. Glutamate-induced alterations in Ca2+ signaling are modulated by mitochondrial Ca2+ handling capacity in brain slices of R6/1 transgenic mice. Eur. J. Neurosci. 32, 60–70; 0.1111/j.1460-9568.2010.07268.x (2010).

26. Naia, L. et al. Histone deacetylase inhibitors protect against pyruvate dehydrogenase dysfunction in huntington’s disease. J. Neurosci. 8:37(10):2276-2794; 10.1523/JNEUROSCI.2006-14.2016 (2017).

27. Naia, L. et al. Comparative Mitochondrial-Based Protective Effects of Resveratrol and Nicotinamide in Huntington’s Disease Models. Mol. Neurobiol. 54(7):5385-8399; 10.1007/s12035-016-0048-3. (2017).
